# Does vitamin D status track through adolescence?^1^

**DOI:** 10.3945/ajcn.115.112714

**Published:** 2015-09-09

**Authors:** Machuene A Poopedi, Shane A Norris, Lisa K Micklesfield, John M Pettifor

**Affiliations:** 2 MRC/Wits Developmental Pathways for Health Research Unit, Department of Paediatrics and; 3 Division of Orthopaedic Surgery, Faculty of Health Sciences, University of the Witwatersrand, Johannesburg, South Africa

**Keywords:** 25-hydroxyvitamin D, *z* scores, tracking, South Africa, adolescence, vitamin D status

## Abstract

**Background:** To our knowledge, no studies have reported on the long-term variability of vitamin D status in adolescents.****

**Objective:** To determine whether tracking of vitamin D status occurs in healthy adolescents, we assessed the variability of 25-hydroxyvitamin D [25(OH)D] every 2 y over a 10-y period in a longitudinal cohort of adolescents living in Johannesburg, South Africa (latitude 26°S).

**Design:** Healthy adolescents who had blood samples available on ≥3 occasions between 11 and 20 y of age were included in the study. Of the cohort of 504 children, 99 met the criteria. The mean 25(OH)D concentration at each time point was measured, and the individual 25(OH)D *z* scores based on year 11 values were used as the reference. All 25(OH)D concentrations for a subject were measured in a single assay.

**Results:** No significant correlation was found between 25(OH)D in the earlier and later years of adolescence, although significant correlations were found between year 11 and year 13 (*r* = 0.71, *P* < 0.0001) and between years 15, 17, and 20 (*r* ≥ 0.65, *P* < 0.0001). The percentage of adolescents whose 25(OH)D concentration changed by >20 nmol/L from year 11 was calculated for all age groups: 12% of the cohort had a change of >20 nmol/L at 13 y of age compared with 46% at 20 y of age. Just more than one-half (53%) of the cohort changed their category of vitamin D status between the ages of 11 and 20 y, and one-third of adolescents changed from being replete to insufficient over the same period.

**Conclusions:** The data suggest that the measurement of 25(OH)D at a single time point does not reflect the long-term vitamin D status of an adolescent. These findings may cast doubt on the veracity of those studies that suggest an association of vitamin D status with various disease states in which vitamin D status was measured only once.

See corresponding editorial on page 985.

## INTRODUCTION

The classic role of vitamin D in calcium and bone homeostasis and its role in cell differentiation, function, and proliferation are well known (1). Recently, attention has focused on the high prevalence of low vitamin D status in many communities around the world (2, 3), including countries at both low and high latitudes (4). Many studies have reported associations between vitamin D status and the prevalence of diseases such as asthma, autoimmune diseases, and some cancers. However, in most of these studies the vitamin D status was assessed by the single measurement of 25-hydroxyvitamin D [25(OH)D] (1, 5–7). It is possible that the values obtained may not reflect the individuals’ vitamin D status over time, because they may vary as a consequence of changes in the factors that influence vitamin D intake or vitamin D formation in the skin (8–10). Few studies have assessed the change in vitamin D status in participants over time. In a Norwegian study, in which 25(OH)D was measured 18 y apart in >2700 adults, the correlation between the two 25(OH)D *z* scores was 0.42, and ∼46% of subjects had a <10 nmol/L change in 25(OH)D over the 18 y (11). In a further analysis, the authors selected 94 adults from the initial sample, who had 25(OH)D concentrations measured every 3 mo for 1 y: ∼71% had a change in 25(OH)D of <10 nmol/L from baseline and 95% had a change in 25(OH)D of <20 nmol/L at the end of the tracking (11). A Canadian study that examined vitamin D status over a period of 10 y reported that ∼50% of subjects had a change in 25(OH)D >20 nmol/L over the 10-y period (12).

Studies on vitamin D status in South African populations have been conducted in adults, and all of these studies measured 25(OH)D concentrations at one time point (13–16). In South Africa, vitamin D intake from the diet is limited because few foods are fortified with vitamin D; thus, the population is almost totally dependent on skin synthesis of vitamin D. The use of vitamin D supplements is uncommon in most of the population (17, 18). During adolescence, major changes generally occur in an individual’s lifestyle, with possible reductions in physical activity and the time spent outdoors and increases in skin coverage by clothing as the adolescents grow older (19, 20). Thus, one might expect that there would be major changes in 25(OH)D concentrations during this period; however, few data exist on vitamin D status in this age group, and, to our knowledge, no studies have reported on the long-term variability in vitamin D status. Therefore, we prospectively studied the variation in serum 25(OH)D concentrations over a 10-y period in a longitudinal cohort of adolescents living in Johannesburg, South Africa.

## METHODS

### Subjects

The subjects were healthy urban South African children living in the greater Johannesburg metropolitan region (latitude 26°S), who formed part of the Bone Health subcohort of the longitudinal Birth to Twenty cohort. The Birth to Twenty cohort’s characteristics, recruitment, and exclusion and inclusion criteria are reported in previous publications (17, 21, 22). Briefly, this cohort of children has been followed since their birth in 1990 and consisted initially of 3273 black and white singleton deliveries that occurred within the greater Johannesburg metropolitan area over a 6-wk period. The Bone Health cohort, which is representative of the larger Birth to Twenty cohort, consists of 504 black and white children who, since the age of 9 y, have been intensively studied annually to investigate factors influencing bone mass accrual and fracture risk during adolescence. Children returned for annual visits as close to the birth month as possible over a period of 10 y. The study protocol was approved by the Committee for Research on Human Subjects of the University of the Witwatersrand, Johannesburg (approval no. M980810), and a parent/guardian gave written informed consent for their children to participate in the study; participating adolescents gave verbal assent or written consent depending on whether or not they were younger than 18 y of age.

### Selection criteria

Children with metabolic bone diseases or chronic illnesses such as juvenile rheumatoid arthritis, epilepsy, or asthma or who were taking vitamin D supplements or medications that affect bone mass or calcium homeostasis were excluded from the study. Serum 25(OH)D was measured biennially over the 10-y period from 11 to 20 y of age. Only those children who had serum available for the measurement of 25(OH)D on ≥3 occasions over this period were included. Of the initial 504 children, 99 met these criteria.

### Measurement of serum 25(OH)D

Fasting serum samples after separation were analyzed for 25(OH)D by chemiluminescent assay (Diasorin Liason) in a laboratory that participates in an international quality assurance program—the International Vitamin D External Quality Assessment Scheme (DEQAS; London, United Kingdom). Since our participation in DEQAS external quality control, our laboratory received the certificates of efficiency on a yearly basis (i.e., ≥80% of results decreased within 30% of the all-laboratory trimmed mean). The same control and reagent lots were used for the analysis of the samples, and all of the samples—in duplicate—from an individual participant were run in the same assay. The interassay CVs for the low and high controls were 8% and 6%, respectively, and the intra-assay CVs for the low and high controls were 5% and 3%, respectively.

### Statistical analysis

The data were analyzed by using the Statistica software package (version 6; StatSoft). Age, height, weight, BMI, and 25(OH)D concentrations were normally distributed. To assess tracking patterns, 25(OH)D *z* scores were calculated for each participant at each time point by using the mean and SD values of year 11 as the reference value. The changes in 25(OH)D *z* scores and actual 25(OH)D concentrations between baseline and the other years were calculated by subtracting the relevant values of the particular year from the baseline (year 11) *z* scores or actual concentrations. One-factor ANOVA tests were used to analyze differences in mean height, weight, BMI, 25(OH)D concentrations, and their respective mean *z* scores among the 5 age groups. A post hoc test (least-significant difference) was used to determine individual group differences. A *P* value <0.05 was considered statistically significant. Pearson correlation tests were conducted to determine the associations between 25(OH)D values measured at the various time points. The change in 25(OH)D between time points in each subject was divided into 3 categories of <10, 10–20, and >20 nmol/L to assess the 25(OH)D stability over a period of 10 y.

## RESULTS

Mean age, height, weight, BMI, and 25(OH)D concentrations and their respective *z* scores for the 5 measurement years are presented in **Table 1**; the number of subjects varied from 99 at baseline (year 11; 58% males and 42% females) to 76 at the end of the study (year 20; 59% males and 41% females). Significant differences in mean height, weight, BMI, 25(OH)D concentrations, and *z* scores between the respective age groups were shown by one-factor ANOVA (*P* < 0.01). Combining the measurements over all the years (years 11–20; *n* = 423) showed that 5% of the measurements were deficient (<30 nmol/L), 35% were insufficient (30–50 nmol/L), and 60% were replete (>50 nmol/L) in vitamin D. Black children had significantly lower 25(OH)D concentrations than their white peers in early and late adolescence. However, within each ethnic group, the mean 25(OH)D concentration was similar in early and late adolescence. The proportion of visits by each individual over the 10-y study period that fell within the same season was ∼76%.

**TABLE 1:** Mean 25(OH)D concentrations and *z* scores at 5 time points during adolescence^1^

Variable	Year 11: baseline (*n* = 99)^2^	Year 13 (*n* = 82)^3^	Year 15 (*n* = 76)^4^	Year 17 (*n* = 90)^5^	Year 20 (*n* = 76)^6^	*P* (ANOVA)
Age, y	11.6 ± 0.3	13.7 ± 0.2	15.6 ± 0.3	17.7 ± 0.2	20.8 ± 0.6	
Height, cm	143 ± 7.3	155 ± 8.1	162 ± 8.7	165 ± 8.8	167 ± 7.9	<0.0001**
Height *z* score	−0.004 ± 0.10	−0.01 ± 0.10	−0.003 ± 0.10	0.001 ± 0.10	0.01 ± 0.10	1.00 (NS)
Weight, kg	37 ± 8.4	48 ± 11.2	57 ± 12.1	60 ± 12.2	64 ± 14.6	<0.0001**
Weight *z* score	−0.001 ± 0.10	−0.0004 ± 1.0	−0.003 ± 0.10	0.001 ± 0.10	0.003 ± 0.10	0.99 (NS)
BMI, kg/m^2^	18 ± 2.9	20 ± 3.6	22 ± 4.1	22 ± 4.1	23 ± 4.7	<0.0001**
BMI *z* score	−0.02 ± 1.0	−0.001 ± 1.0	−0.005 ± 1.0	−0.004 ± 1.0	−0.01 ± 1.0	0.99 (NS)
25(OH)D, nmol/L	58 ± 5.8*	58 ± 7.7*	55 ± 7.7	60 ± 7.7*	50 ± 7.2	<0.01**
25(OH)D *z* score	0.0001 ± 1.0*	0.05 ± 1.3*	−0.09 ± 1.3	0.19 ± 1.3*	−0.44 ± 1.2	<0.01**

1 All values are means ± SDs. *Significant difference between year 20 and other years [actual 25(OH)D values and 25(OH)D z scores] determined by a post hoc test. **Significant variations in mean height, weight, BMI, 25(OH)D, and 25(OH)D *z* scores by age determined by ANOVA. 25(OH)D, 25-hydroxyvitamin D.

2 *n* = 90 black and 9 white.

3 *n* = 76 black and 6 white.

4 *n* = 69 black and 9 white.

5 *n* = 81 black and 9 white.

6 *n* = 70 black and 6 white.

Seasonal variations in 25(OH)D concentrations were found, which were more marked in white than in black adolescents (73 and 57 nmol/L in summer/autumn and 61 and 53 nmol/L in winter/spring in whites and blacks, respectively).

In a further analysis of the difference between the age groups, a post hoc test (least-significant difference) was used. The 25(OH)D concentrations and *z* scores at year 20 were significantly lower than the values at year 11 (*P* < 0.02), year 13 (*P* < 0.01), and year 17 (*P* < 0.001) but not when compared with year 15.

The correlations between 25(OH)D values at the different time points are presented in **Table 2**. The 25(OH)D concentrations at year 11 correlated significantly with those at year 13 (*P* < 0.0001), but did not correlate with any of the concentrations at other ages. The concentrations at years 15, 17, and 20 correlated significantly with one another (all *P* < 0.0001) but not with those at earlier years.

**TABLE 2:** Pearson correlation coefficients between individual participants’ 25(OH)D concentrations obtained in each of the study years^1^

	Year 11 (*n* = 99)	Year 13 (*n* = 82)	Year 15 (*n* = 76)	Year 17 (*n* = 90)	Year 20 (*n* = 76)
Year 11 (*n* = 99)					
* r*		0.706	−0.087	−0.052	−0.151
* P*		<0.0001	0.6	0.7	0.4
Year 13 (*n* = 82)					
* r*	0.706		0.079	0.011	−0.004
* P*	<0.0001		0.6	0.1	0.8
Year 15 (*n* = 76)					
* r*	−0.087	0.079		0.733	0.649
* P*	0.6	0.6		0.0001	<0.0001
Year 17 (*n* = 90)					
* r*	−0.052	0.011	0.733		0.701
* P*	0.7	0.1	<0.0001		<0.0001
Year 20 (*n* = 76)					
* r*	−0.151	−0.004	0.649	0.701	
* P*	0.3	0.9	<0.0001	<0.0001	

1 25(OH)D, 25-hydroxyvitamin D.

A multiple regression analysis (data not shown) was performed to determine whether covariates such as puberty, body composition, ethnicity, sex, or season had an effect on 25(OH)D, but no association was found.

**FIGURE 1:**
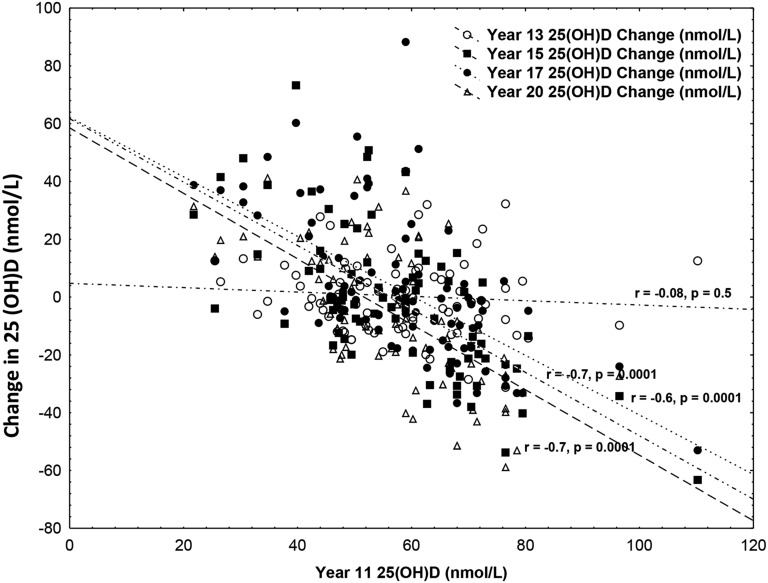
Change over the 10-y study in 25(OH)D concentrations from values at age 11 y. 25(OH)D, 25-hydroxyvitamin D.

The associations between 25(OH)D at age 11 y and changes in 25(OH)D between year 11 and the other ages are presented in **Figure 1**. No significant association was found between 25(OH)D in year 11 and the change in 25(OH)D between year 11 and year 13; however, 25(OH)D concentrations in year 11 were inversely associated with the changes in concentrations between year 11 and all other years.

The percentage of adolescents who had changes in 25(OH)D concentrations of ≤10, 11–20, or >20 nmol/L from baseline to each time point are presented in **Table 3**. Just more than half (57%) of 25(OH)D values at year 13 changed by ≤10 nmol/L from year 11, and only 12% of the cohort had a change in value of >20 nmol/L over these 2 ages. However, at the other time points, ∼40% of the cohort had a change of >20 nmol/L.

**TABLE 3:** Subjects with a change in 25(OH)D ≤10, 11–20, or >20 nmol/L between baseline (11 y of age) and the other time points^1^

	Change in 25(OH)D, *n* (%)		
Variable	≤10 nmol/L	11–20 nmol/L	>20 nmol/L
Year 13 change from year 11 (*n* = 82)	47 (57)	25 (31)	10 (12)
Year 15 change from year 11 (*n* = 76)	28 (37)	17 (22)	31 (41)
Year 17 change from year 11 (*n* = 90)	15 (17)	41 (46)	34 (38)
Year 20 change from year 11 (*n* = 76)	18 (24)	23 (30)	35 (46)

1 25(OH)D, 25-hydroxyvitamin D.

The percentages of adolescents who maintained or changed their vitamin D status between year 11 and year 20 are shown in **Table 4**. Just more than half (53%) of the cohort changed their vitamin D status over the 2 time points, and 33% of the adolescents changed from a replete to an insufficient status over the 10-y period. Just less than one-third (30%) of adolescents remained vitamin D replete between year 11 and year 20.

**TABLE 4:** Comparison of the vitamin D status at baseline (year 11) and in the past year (year 20) of measurement

Vitamin D status category	Sample, %
Vitamin D insufficient at year 11 and at year 20	17
Vitamin D replete at year 11 and year 20	30
Vitamin D insufficient at year 11 and vitamin D replete at year 20	20
Vitamin D replete at year 11 and vitamin D insufficient at year 20	33

## DISCUSSION

The period of adolescence is generally associated with changes in lifestyle, which are often associated with a reduction in outdoors physical activity because of the tendency of playing indoors with computers or watching television (19, 20). These changes predisposed this age group to reduced cutaneous synthesis of vitamin D and thus to alterations in circulating 25(OH)D concentrations, as has been noted in the current study.

Studies in adolescence and adults have reported an association between vitamin D status and various disease states, but 25(OH)D concentrations were measured at one time point only (3, 23, 24). For example, in a retrospective study by Kao et al. (25), vitamin D deficiency was found to be associated with high blood pressure in obese children, yet the vitamin D status was measured only at the time of assessment of the blood pressure. In another case-control study, low serum 25(OH)D appeared to be related to asthma in children, but the conclusions were made based on a single 25(OH)D measurement at the time of assessment (26). Because vitamin D status is influenced by factors that affect skin production of vitamin D (8, 27–29), and also dietary patterns (30) and body composition (31, 32), we hypothesized that a single measurement of 25(OH)D in healthy adolescent children might not reflect vitamin D status over a prolonged period of time. In this study, we have shown that nearly 50% of participants had a change in 25(OH)D of ≥20 nmol/L between 11 and 20 y of age. Furthermore, only one-third of adolescents were vitamin D replete at both 11 and 20 y of age. From a positive perspective, 20% of the cohort who were vitamin D insufficient at 11 y of age were replete at 20 y.

We found that adolescents who had the highest 25(OH)D concentrations at 11 y of age had the largest decrease in 25(OH)D at 15, 17, and 20 y of age, whereas those with low baseline values had a greater increase at the other time points. This suggests that, over time, there is a tendency for 25(OH)D concentrations to regress toward a mean.

Although an individual’s serum 25(OH)D concentrations correlated over relatively short periods of time in early and late adolescence, no significant correlation was found over a longer period; thus, individual participant levels in years 11 and 13 did not correlate with those in years 15, 17, or 20.

Data on vitamin D tracking is still lacking, and only 2 studies in adults have been published, which have conflicting results. In one study, the tracking of vitamin D over a short period (12 mo) was shown to have strong positive correlations between months of tracking (11), whereas poor 25(OH)D tracking was shown over a long period (between 5 and 10 y) in the other study (12). In the current study, tracking was present in early and late adolescence, but not throughout adolescence. We found no good explanation for this dichotomy in results between early and late adolescence; however, it was unlikely to be due to laboratory variations because all samples from a single participant were measured in the same assay. We do acknowledge that only a relatively small proportion of the cohort (99 of 504 participants) was eligible for enrollment into the study because of the requirement to have available blood samples on 3 different occasions over the study period, which could have introduced bias. To better understand the association between vitamin D status and disease states, more longitudinal studies are required to document the pattern of vitamin D tracking in the participants and the factors that influence it. It is possible that the tracking of vitamin D status is least apparent during periods of major lifestyle changes, as occurs in adolescence or in regions where seasonal variations are large.

In conclusion, in our study of South African adolescents, no association was found between 25(OH)D values measured in early and later adolescence. Furthermore, nearly 50% of participants had a change in 25(OH)D of >20 nmol/L over the 10-y study. Thus, tracking was restricted to relative short periods at the beginning and end of adolescence. The current study suggests that a single measurement of 25(OH)D may not accurately reflect the vitamin D status of an individual over a prolonged period of time. These results cast doubt on the veracity of reports that suggest that vitamin D status (as measured on a single occasion) may influence long-term disease outcomes.
